# Current Diagnostic and Therapeutic Challenges in Superficial Venous Thrombosis

**DOI:** 10.3390/medicina60091466

**Published:** 2024-09-06

**Authors:** Ana-Maria Balahura, Adrian-Gabriel Florescu, Teodora-Maria Barboi, Emma Weiss, Daniela Miricescu, Ciprian Jurcuț, Mariana Jinga, Silviu Stanciu

**Affiliations:** 1 Department of Cardiology, “Carol Davila” University of Medicine and Pharmacy, “Prof. Dr. Theodor Burghele” Clinical Hospital, 010024 Bucharest, Romania; ana-maria.balahura@umfcd.ro; 2 Dr. Carol Davila University Central Military Emergency Hospital, 010825 Bucharest, Romania; adrian-gabriel.florescu@rez.umfcd.ro; 3 Emergency Institute of Cardiovascular Diseases Prof. Dr. C.C. Iliescu, 022328 Bucharest, Romania; teodora-maria.barboi@rez.umfcd.ro; 4 Department of Internal Medicine, “Carol Davila” University of Medicine and Pharmacy, Clinical Emergency Hospital Bucharest, 050474 Bucharest, Romania; emma.weiss@umfcd.ro; 5 Department of Biochemistry, Faculty of Dentistry, “Carol Davila” University of Medicine and Pharmacy, 050474 Bucharest, Romania; 6 Dr. Carol Davila University Central Military Emergency Hospital, “Carol Davila” University of Medicine and Pharmacy, Calea Plevnei 134, 010825 Bucharest, Romania; mariana.jinga@umfcd.ro (M.J.); silviu.stanciu@umfcd.ro (S.S.)

**Keywords:** superficial venous thrombosis, thrombophlebitis, venous thromboembolism, duplex ultrasound, anticoagulation, surgery, complications

## Abstract

Superficial venous thrombosis (SVT) is a fairly common disorder, characterized by the formation of thrombi inside superficial veins, with or without an associated inflammatory reaction. Its evolution is frequently self-limited. However, serious complications may change this clinical course with extension to deep vein thrombosis (DVT) and pulmonary embolism (PE). SVT shares similar risk factors with DVT and is frequently associated with the presence of varicose veins. However, the occurrence of non-varicose veins could conceal risk factors such as malignancies, thrombophilia, or Buerger’s disease. While the clinical diagnosis is generally straightforward, additional diagnostic evaluations are often necessary. Duplex ultrasound (DUS) is an invaluable tool that provides the location of SVT, the proximity to the sapheno–femoral junction, and the clot length, all of which influence the decision for optimal management. The treatment of SVT should be symptomatic, pathogenic (limiting the extension of thrombosis), and prognostic (to prevent complications). There are several guidelines that provide recommendations, and despite the need for more consensus and for further studies, the treatment of SVT should be mainly medical, including anticoagulation in specific clinical situations and symptom relief, with invasive treatment in a minority of cases. Initiation, intensity, and length of anticoagulant treatment should be based on the eventual risk of progression to DVT or PE, which can be high, intermediate, or low, based on the location of SVT and the clot length. Our review summarizes the evaluation and proper management of SVT and highlights the importance of a shared decision within the heart team regarding this condition in order to prevent further complications.

## 1. Introduction

Superficial venous thrombosis (SVT) is a common thrombotic disorder characterized by the formation of a thrombus in a superficial vein, commonly associated with an inflammatory reaction. Its clinical course is, in most cases, self-limited; however, in some patients, serious or even fatal complications may change this clinical course. Although not a rare medical condition, data are scarce regarding predisposing risk factors, prevalence of concomitant deep vein thrombosis (DVT) and/or pulmonary embolism (PE), and therapeutic strategies in order to prevent its complications. However, clinicians should be aware of what is currently known in term of risk factors, complications, and existing therapies in order to organize a complete work up (clinical, imaging and laboratory) and a proper therapeutic strategy. The aim of this narrative review is to summarize the existing available data regarding diagnosis, treatment, and potential complications of SVT.

## 2. Definition and Epidemiology

SVT is characterized by the formation of thrombi within the superficial veins, leading to partial or complete occlusion of the lumen, which may occur with or without an accompanying inflammatory reaction along the venous path. SVT affects 3 to 11% of the general population [1]. The veins of the lower limbs are usually involved; however, SVT can also be found in other sites. In 60–80% of SVT cases, the great saphenous vein is involved, while the small saphenous vein is implicated in 10–20% of cases [2]. A small retrospective study revealed that isolated brachiocephalic vein and superior vena cava thrombosis occur frequently enough among hospitalized patients to warrant serious consideration, particularly in those with cancer, central venous access lines, or both. Within the studied group, 74% of patients were diagnosed with cancer, and 65% had central venous access lines [3].

Moreover, a community-based study found that the annual incidence of SVT was nearly 6 per 1000 patients, roughly three times higher than that of DVT. Despite this, SVT is associated with DVT in 18.1% of patients and linked to more severe complications, such as PE, in 6.9% of cases. These findings were highlighted in a meta-analysis that examined 21 studies (4358 patients) for DVT prevalence and 11 studies (2484 patients) for PE prevalence in those with SVT [2].

The prevalence of concurrent acute DVT varies widely, influenced by differences in study design, patient characteristics, symptomatic presentation, type of SVT, and whether patients are in an inpatient or outpatient setting. A significant prospective observational study (the POST study) reported that, within the first three months of follow-up, 10.2% of patients with SVT experienced thromboembolic events, including DVT, PE, DVT progression, or recurrent SVT [4].

A study on outpatients diagnosed with SVT reported a 13% incidence of acute DVT. However, this rate varied significantly: 6.3% among patients with varicose veins, 33% among patients without varicose veins, and 40% in individuals with a prior episode of DVT [5].

Regarding the long-term (>3 months) thromboembolic risk following an episode of SVT, persistent venous stasis and additional risk factors such as thrombophilia, autoimmune disease, and malignancies appear to be associated with recurrent thromboembolic events [6].

In a study of 147 patients with significant SVT treated with tinzaparin, recurrent thromboembolic events occurred in 10.2% of those receiving variable doses of the medication. This rate was lower among patients on intermediate doses administered for 3 months. Additionally, extensive SVT emerged as an independent predictor for recurrent thromboembolic events, with a hazard ratio of 5.94 (95% confidence interval, 2.05–17.23; *p* = 0.001) [7]. Furthermore, in a small study involving 171 patients with SVT diagnosed through ultrasonographic compression, DVT was identified in 24.6% of cases, while PE was reported in 4.7% of the patients [6].

However, in a retrospective cohort study, it was suggested that spontaneous SVT in the leg is a risk factor for DVT (occurred in 2.7% of all SVT patients as compared with 0.2% in the control group) but is less predictive for other embolic events, including PE, acute coronary events, or ischemic stroke over a 6-month follow-up period [8].

In conclusion, due to the small sample sizes and significant variability in anticoagulant treatments across existing studies, establishing a clear correlation between SVT and major thromboembolic events remains challenging. Therefore, there is a need for larger studies with optimized therapeutic strategies for SVT to provide more definitive insight.

## 3. Etiology and Risk Factors

Risk factors for SVT fall within common prothrombotic conditions (Table 1), including recent surgery or trauma, prolonged immobilization, inherited or acquire thrombophilia, active cancer, infectious diseases, oral contraceptive use, obesity, and cardiac or respiratory failure. However, varicose veins are the primary risk factor for the development of SVT, documented in up to 80% of affected patients [2,9].

In patients with varicose veins, the inflammatory reaction is frequently triggered by minor trauma. On the other hand, cancer, thrombophilia, and autoimmune disorders (Behcet’s disease, Buerger’s disease) may cause SVT in morphologically normal veins. Data from small, observational studies show a high prevalence of these risk factors in patients with SVT in non-varicose veins, including thrombophilia (47.6% patients), malignancy (4.76% patients), and other non-malignant systemic condition (9.52% patients) [10].

The presence of any intravenous catheter has the potential to cause venous thrombosis. A systematic review identified 425 catheter-related thromboses among 5636 patients with malignancy (7.5%) across five trials and seven prospective studies. Factors associated with an increased risk of catheter-related thrombosis, as determined by multivariate logistic regression, included the use of peripherally inserted central catheters, improper positioning of the catheter tip, and previous history of DVT [11].

The association of spontaneous venous thromboembolism with occult malignancy is well established. A single-center, retrospective study analyzing 276 patients with SVT showed that cancer was the strongest determinant of concurrent DVT/PE. The prevalence of malignancy was 8.7%, commonly due to breast and urinary tract cancer, comprising 4.2% of those with isolated SVT and 18.8% of those with SVT and coexistent DVT/PE (*p* < 0.001) [12]. Furthermore, data from the large prospective observational INSIGHTS-SVT study, which included patients with acute isolated SVT, demonstrated that malignancy significantly raises the risk of VTE associated with SVT at both 3 and 12 months, suggesting that extended anticoagulation may be beneficial for these patients. The INSIGHTS-SVT trial found that, among 1151 patients with SVT, 6.7% either had active cancer at baseline or were diagnosed with cancer during the one-year follow-up period. Symptomatic VTE was notably more prevalent in cancer patients compared to non-cancer patients (13% vs. 5.4%) after a three-month follow-up. Additionally, cancer increased the risk for DVT or PE (HR 3.9, 95% CI 1.3–11.8), hospitalization due to VTE (HR 11.0, 95% CI 2.5–49.0), and relevant bleeding (3.9% vs. 1.3%) [13]. Taking into account all of this data, the identification of a possible associated cancer is one of the most important steps in the management of SVT, especially in patients without risk factors. As we do not have specific recommendations for cancer screening in patients with SVT, we could, for the moment, use the information from existing guidelines for VTE [14].

Patients with thrombophilia have a significantly increased incidence of superficial vein thrombosis (SVT), with the risk being elevated two-fold to six-fold. Notably, approximately 40% of individuals with factor V Leiden and up to 15% of those with deficiencies in protein C or S are affected by SVT. Also, patients with recurrent SVT are more likely to have anticardiolipin antibodies and elevated factor VIII levels. However, the association of the prothrombin G20210A mutation with SVT remains controversial [1,15].

However, data on the risk factors for SVT in patients with or without varicose veins are otherwise limited. SVT as a second event after DVT tends to be more frequent in older and overweight or obese patients—BMI (body mass index) greater than 25 kg/m^2^—and the location of venous thrombosis (proximal or distal) is an indifferent factor of occurrence [1].

**Table 1:** Etiology and risk factors [1,2,9,10,11,14,15].

Etiology and risk factors
History of venous thromboembolism
Malignancy
Recent surgery or trauma
Immobilization
Inherited or acquired thrombophilia
Use of oral contraceptives
Infectious disease
Obesity
Cardiac of respiratory failure
Varicose veins
Burger’s disease
Natural coagulation inhibitory deficiency
Mutation factor V Leiden
Antiphospholipid syndrome
Increased levels of factor VIII

## 4. Diagnosis

Although the clinical diagnosis is generally straightforward, further diagnostic tests are essential to assess the full extent of thrombosis and to identify any potential thromboembolic complications. In individuals exhibiting a high index of suspicion (patients with venous catheters, venous punctures, or varicose veins) the presence of compatible symptoms is suggestive of SVT.

The diagnosis of SVT is made in a clinical setting by recognizing the presence of a firm, thickened, thrombosed vein alongside inflammatory signs (pain, swelling, and redness). Thrombosis in the saphenous veins and their tributaries is the most common occurrence, followed by the involvement of veins of the upper extremities (cephalic and basilic vein). Thrombosis in superficial veins in other parts of the body is uncommon [16]. 

After clinical assessment, a comprehensive imaging evaluation is paramount to determine its severity and identify any additional local or systemic complications. This aspect is highlighted as a Class I recommendation in the European Society for Vascular Surgery (ESVS) 2021 Clinical Practice Guidelines on the Management of Venous Thrombosis [4]. It is suggested by some authors that SVT occurring in the great saphenous vein within 3 cm from the sapheno–femoral junction carries a risk of PE comparable to that of DVT [16]. Furthermore, SVT involving the arches of the sapheno–femoral/sapheno–popliteal junctions has an elevated risk of recurrence resembling that of DVT [7]. In these settings, duplex ultrasound (DUS) is an invaluable tool for providing the location of SVT (varicose veins, great saphenous vein, small saphenous vein), the proximity to the sapheno–femoral junction (≤3 cm), and the clot size (length > 5 cm), all parameters that influence the decision regarding the choice of optimal medical or surgical treatment. Similar to the lower limbs, the presence of compressibility of the upper limb deep veins during DUS can safely exclude DVT. The primary criteria for diagnosing thrombosis include the vein’s non-compressibility, abnormal flow patterns such as flow reversal, and the presence of a visible intraluminal thrombus, with sensitivity and specificity ranging from 80% to 100%. 

A systematic review of 17 studies concluded that compression ultrasonography is a viable alternative to standard contrast venography also in upper extremity vein thrombosis. The review found that the summary estimates for sensitivity were 97% for compression ultrasonography, 84% for duplex ultrasound, and 81% for duplex ultrasound with compression. Specificity estimates were 96% for compression ultrasonography, 94% for duplex ultrasound, and 93% for duplex ultrasound with compression [17]. The chance of a false-positive study is very low. However, non-occlusive mural thrombus and thrombus in the proximal subclavian or brachiocephalic veins may not be adequately visualized, stressing the need for other imaging tools in order to confirm the diagnosis [17].

Point-of-care ultrasound (POCUS) is increasingly utilized in the intensive care unit, emergency department, medical wards, and outpatient settings to evaluate the proximal lower extremity venous system. There are two primary types of POCUS exams for diagnosing DVT: “2-point” and “3-point” exams. The “2-point” POCUS technique examines the common femoral vein and the popliteal vein. By contrast, the “3-point” technique includes additional scanning of the femoral vein in the proximal thigh, covering the sapheno–femoral junction, the proximal and mid-distal femoral vein, and the popliteal vein. Research indicates that POCUS may offer diagnostic accuracy comparable to venography or vascular lab-performed DUS for detecting proximal lower extremity DVT, making it an invaluable tool in routine clinical practice [18]. 

As discussed above, a recent meta-analysis suggested that patients with SVT exhibit a significant risk of concomitant DVT or PE at the time of SVT diagnosis [2]. There are no studies directly comparing the accuracy and effectiveness of various diagnostic methods for DVT. However, DUS has become the preferred method due to its low cost, diagnostic effectiveness, and minimal risk to patients [19]. Therefore, DUS should be performed in all patients with SVT in order to evaluate the presence of a possible DVT. Also, in the settings of high clinical susceptibility for PE, computed tomography angiography or ventilation/perfusion scintigraphy can confirm the diagnosis alongside echocardiography, which can identify acute RV dysfunction. The diagnosis of DVT or PE in patients with SVT is important not only as a prognostic factor, but it also has therapeutic implications. By contrast, phlebography lacks sufficient accuracy and an opportune risk–benefit ratio for routine use in SVT [19] cases and is not considered a first-line imaging modality.

Searching for the etiology of SVT is one of the second steps in the diagnostic algorithm. The varicose veins are evident at clinical examination and at ultrasonography. The evaluation of genetic or acquired thrombophilia should be guided by the family history of venous thromboembolic events and other coexisting clinical elements. Very rare causes (i.e., Buerger’s diseases, Behcet’s disease) have a particular clinical presentation and are evident in most of the patients. The identification of a possible cancer is challenging and should be guided by the sex and age of the patients, specific risk factors for neoplasia (i.e., smoking for certain cancers), and symptoms suggestive of neoplastic diseases or different clinical elements regarding superficial vein thrombosis (recurrent episodes, occurrence in non-varicose veins). 

## 5. Management

SVT is an acute, intensely symptomatic disease that interferes with quality of life and, through its complications, can even pose a vital threat. The treatment of SVT should be symptomatic, pathogenic (limiting the extension of thrombosis), and prognostic (to prevent recurrence or evolution to DVT or PE). 

At the moment, it is necessary to understand that treatment of SVT should be mainly medical, including anticoagulation in specific clinical situations and symptom relief, with interventional or surgical treatment in a minority of cases [20]. 

### 5.1. Anticoagulation

The decision for initiation, intensity, and length of anticoagulation is based on the eventual risk of progression to DVT or PE, which can be high, intermediate, or low [21].

(A) Superficial vein thrombosis adjacent to the deep vein system (<3 cm from sapheno–femoral junction) (high risk).

Superficial vein thrombosis adjacent to the deep vein system, more precisely, <3 cm from the sapheno–femoral junction, is a high-risk situation and therefore it is recommended to be treated with a minimum of 6 weeks of full anticoagulation [4,22]. Given the high risk of progression to thromboembolic vein disease (variable incidence between 10 and 70% throughout the literature), this entity has traditionally been excluded from trials with SVT and treated with full-dose anticoagulation, despite the lack of direct evidence from studies [4,23]. In the 2023 Society for Vascular Surgery, American Venous Forum, and American Vein and Lymphatic Society clinical practice guidelines for the management of varicose veins of the lower extremities, this recommendation is mentioned as a consensus statement [4]. However, there is room for discussion in these particular cases. For instance, Prandoni et al. conducted a study based on the RIETE registry, which enrolled patients with confirmed VTE. They retrospectively studied the evolution of patients with isolated SVT within 3 cm from the saphenous–femoral junction, with 60.7% of them having been treated with full-dose of low molecular weight heparins (LMWH) or fondaparinux followed by vitamin K antagonists or direct oral anticoagulants and 39.3% of them having been treated with intermediate doses of LMWH or prophylactic dose of fondaparinux. The results showed that subtherapeutic anticoagulant doses are very likely to be as effective as high doses, but with a lower risk of hemorrhagic complications [24]. As the authors of the study also mentioned, a randomized controlled trial comparing these two approaches is necessary to decide the optimal therapeutic strategy in SVT adjacent to the deep vein system. In our opinion, in this situation, several additional risk factors, such as sex, age, known thrombophilia, previous venous thrombotic events, concomitant cancers, or SVT of non-varicose veins, could be instrumental in deciding the dose and the duration of anticoagulant treatment.

(B) Main saphenous trunks and tributaries above the knee, >3 cm from the sapheno–femoral junction and at least 5 cm in length (intermediate risk)

The strongest recommendation from the ESVS 2021 Clinical Practice Guidelines on the Management of Venous Thrombosis, the 2023 Society for Vascular Surgery, American Venous Forum, and American Vein and Lymphatic Society guidelines for the management of main saphenous trunks and tributaries above the knee, >3 cm from the sapheno–femoral junction or sapheno–popliteal junction, and at least 5 cm in length, is the use of fondaparinux 2.5 mg subcutaneously (sc) daily for 45 days, whether or not associated with varicosities. This indication is supported by the CALISTO trial and two systematic reviews (Table 2) [4,22,25,26].

The SURPRISE study was published in 2016 and proved that a low dose of rivaroxaban (10 mg od for 45 days) is non-inferior compared to fondaparinux 2.5 mg sc in preventing embolic complications and has a similar rate of complications. The results of this trial are auspicious for everyday practice, as it offers an alternative to treatment with fondaparinux, which is a more expensive treatment and needs daily subcutaneous injections [27,28]. However, in a large meta-analysis, the authors concluded that rivaroxaban 10 mg needs further evaluation for this indication, as the SURPRISE trial had some limitations: the sample size was not powered to prove non-inferiority and the researchers observed a non-statistical increase in the incidence of non-major bleedings, which should be further investigated [29].

As far as low-molecular-weight heparins (LMWHs) are concerned, although proved effective in alleviating symptoms and preventing extension, scientific societies recommend in their guidelines against using them in SVT of the main venous trunks as the available evidence does not prove a significant effect in preventing VTE, based on the heterogeneity and lack of statistical significance found by Di Nisio et al. throughout studies included in their meta-analysis [22,29].

However, fondaparinux is an expensive treatment, not always and everywhere available, whereas the SURPRISE trial with rivaroxaban had some limitations that await to be clarified in further studies; therefore, fondaparinux seems to be the optimal treatment for SVT partly because it is supported by the most rigorous scientific evidence. Therefore, in our opinion, a more convenient alternative should be available in therapeutic recommendations. 

In the ESVS 2021 Clinical Practice Guidelines on the Management of Venous Thrombosis, there is Class IIa recommendation that a regimen with an intermediate dose of LMWH for 45 days (Table 2) could be used as an alternative to fondaparinux [4]. The guidelines on the investigation and management of venous thrombosis at unusual sites, realized by the British Society for Hematology in 2012 and reviewed in 2022, also offer the alternative to treat with prophylactic doses of LMWH for a minimum 30 days, mentioning that it is an unlicensed indication [30]. Moreover, at this point, there is some evidence that in presence of some certain thrombotic risk factors, such as recurrent SVT, SVT related to thrombophilia, or malignancy, it would be beneficial to prescribe anticoagulants for a longer period than the initial 30–45-day period (up to 3 months; Class IIb indication) [4] (Table 3).

Karathanos et al. recently published a study supporting the use of LMWH for this indication: they performed a pooled analysis from two prospective studies, which assessed the usage of an intermediate dose of tinzaparin (75% of the therapeutic dose) in SVT patients. The results showed that the intermediate dose of tinzaparin for 30 days seems to be a safe and effective way to treat SVT. Another interesting finding was that the duration of treatment was not related to the recurrence of venous thromboembolism; the only risk factor significantly associated with recurrence was the length of thrombus at initial presentation [31].

**Table 2:** RCTs, systematic reviews, and meta-analyses supporting the use of anticoagulants in SVT [26,28,29,32,33,34,35,36,37,38,39,40,41,42].

Anticoagulant	Studies	No. Patients	Conclusions
Fondaparinux	CALISTO trial, 2010 (RCT)	3002	Fondaparinux 2.5 mg sc daily is superior to placebo
	Duffet et al., 2019 (meta-analysis)	6862	Fondaparinux is associated with the lowest VTE event rate during follow-up between patients with SVT treated with NSAIDs/anticoagulant therapies/surgical therapies/observation or placebo
	Di Nisio et al., 2018 (systematic review)	7296	This study supports fondaparinux in prophylactic dose for 45 days as efficient in superficial vein thrombosis
Rivaroxaban	SURPRISE trial, 2017 (RCT)	472	Rivaroxaban (10 mg od) is non-inferior to fondaparinux (2.5 mg od sc)
	Single center retrospective case record review at King’s College Hospital, 2021	54	Rivaroxaban is effective and safe for the treatment of SVT
	Rivaroxaban compared to placebo for the treatment of leg superficial vein thrombosis: A randomized trial, 2020 (RCT)	85	The conclusion derived from the study is that rivaroxaban is effective in the treatment of SVT, although based on limited data
LMWH	STEFLUX trial, 2012 (RCT)	664	A 30-day regimen of an intermediate dose of parnaparin is more effective than both a 30-day regimen of a prophylactic dose and a 10-day regimen of an intermediate dose.
	REVETR study, 2014 (RCT)	68	Patients were assigned to either a prophylactic dose of dalteparin (5000 IU od) or to a double dose (10,000 IU od); the conclusion was that the dosage of anticoagulant does not impact the rate of thrombus resolution
	Rathbun et al., 2012 (RCT)	72	Dalteparin (intermediate dose) is superior to the NSAID ibuprofen
	Prandoni et al., 2005 (RCT)	164	Therapeutic dose of nadroparin, administered for 1 month in patients with SVT, is not superior to prophylactic dose
	Lozano et al., 2003 (RCT)	84	No statistically significant differences between enoxaparin (1 mg/kg b.i.d. for the first week, then 1 mg/kg od for 3 weeks) and saphenofemoral disconnection; however, enoxaparin group had socioeconomic advantages
	STENOX group, 2003 (double-blind trial)	427	Comparison between enoxaparin 40 mg od, enoxaparin 1.5 mg/kg od, oral tenoxicam, and placebo for 8–12 days; there was observed a benefit of the active treatment over placebo, but the results were not statistically significant
	Titon et al., 1994 (multicenter, randomized, open trial)	117	Calcium nadroparin proved a better efficacy in improving symptoms and signs comparative to naproxen; a higher effect of nadroparin on repermeabilization of the thrombosed vein was also observed, but the result was not statistically significant
	Gouveia et al., 2018 (retrospective cohort)	60	Patients were treated with enoxaparin 40 mg od (and 80 mg od for obese patients > 100 kg); the findings support LMWH usage in SVT

**Table 3:** Summary of the available recommendations in international guidelines regarding management of SVT [4,22,30,43].

Guidelines	Recommendations
ESVS 2021 Clinical Practice Guidelines on the Management of Venous Thrombosis	A 45-day regimen of anticoagulation is recommended in the following situations (Class I B): Superficial vein thrombosis ≥ 3 cm away from the deep venous system and ≥ 5 cm in length: Fondaparinux 2.5 mg sc once daily (Class I B) Intermediate dose of LMWH (Class IIa B) Superficial vein thrombosis < 3 cm to the deep venous system: Anticoagulation at therapeutic doses (Class I C) Special considerations: A three-month regimen of anticoagulant treatment should be taken into account if high-risk clinical and/or anatomical features are present (Class IIb C) Surgical ablation of incompetent superficial veins is recommended after the acute phase has resolved, typically no sooner than three months following the most recent thrombotic event
The 2023 Society for Vascular Surgery, American Venous Forum, and American Vein and Lymphatic Society clinical practice guidelines for the management of varicose veins of the lower extremities	SVT of the main saphenous trunks and tributaries above the knee, >3 cm from the SFJ, and >5 cm in length (with or without varicose veins): fondaparinux 2.5 mg s.c. for 45 days or rivaroxaban 10 mg o.d. for 45 days (an appealing option to avoid daily injections) (Class I A). SVT of the main saphenous trunks, <3 cm from the SFJ: anticoagulation at therapeutic doses for 6 weeks (consensus statement) SVT of the saphenous trunks: against use of therapeutic-dose LMWH and NSAIDs (Class I A) Isolated thrombosis of varicose tributaries or limited involvement of the great saphenous vein: phlebectomy is suggested as a viable alternative (Class II B)
Guidelines on the investigation and management of venous thrombosis at unusual sites (British Society for Haematology; last reviewed in 2022)	Confirmed SVT <3 cm to the sapheno–femoral junction: considered for anticoagulant treatment at therapeutic doses (Class 2B) SVT in patients presenting favorable factors for extension, recurrence, or progression: treatment with prophylactic doses of LMWH for 30 days (unlicensed indication) or fondaparinux for 30–45 days (Class 1B) Other patients with SVT: 8–12 days of NSAIDs in the absence of contraindications (Class 1A)
Thrombosis Canada Guide (last revised 2023)	Isolated SVT <3 cm to the sapheno–femoral junction (SFJ) or sapheno–popliteal (SPJ) junction: Full-dose anticoagulation for 3 months Isolated SVT ≥5 cm in length located >3 cm from the SFJ: low-dose fondaparinux (2.5 mg) or rivaroxaban (10 mg o.d.) or prophylactic/intermediate doses of LMWH Isolated SVT <5 cm in length, located >3 cm from the SFJ/SPJ: mainly symptomatic relief by orally or topically administered NSAIDs, compresses (warm or cool), and leg elevation In isolated SVT <5 cm in length, located >3 cm from the SFJ/SPJ, but with intense symptoms or risk factors for progression (for example, history of DVT/PE or SVT, malignancy, pregnancy, hormonal therapy, recent surgery or trauma): low-dose fondaparinux (2.5 mg) or rivaroxaban (10 mg o.d.) or prophylactic/intermediate doses of LMWH

(C) SVT > 3 cm from the sapheno–femoral junction and <5 cm in length (low risk).

This category represents the low-risk spectrum of SVT in the lower limbs. The guidelines of British Society for Haematology and other experts agree that treatment with NSAIDs (non-steroidal anti-inflammatory drugs), either orally or topical, for 7 to 14 days, is an acceptable strategy for these patients [23,30,43]. For example, in the STENOX trial, tenoxicam has been proven to reduce SVT extension or relapse [40]. However, in this approach, there are some important elements to consider: 

-to judge clinically and to search for additional risk factors, such as previous thromboembolic events, malignancy, known thrombophilia, absence of varicose veins, or severe symptoms that could impose the need for systemic anticoagulant treatment;

-DUS re-evaluation should be performed at the end of the NSAID treatment to exclude extension into the deep venous system [23,28,30,33,35,43].

-although the duration of treatment with NSAIDs is generally short, up to 14 days, there are patients with certain co-morbidities that could increase the risk of adverse effects to NSAIDs: recent gastrointestinal bleeding, peptic ulcer, patients with antithrombotic therapy following a myocardial infarction or an elective coronary revascularization, patients at risk of developing exacerbation of their respiratory disease induced by NSAID use, or patients with a history of renal disease [44].

### 5.2. Symptomatic Relief

Symptomatic treatment involves a multi-faceted approach aimed to reduce inflammation and alleviate symptoms. Beneficial interventions could include extremity elevation, warm or cold applications, topical NSAIDs or heparin gel, and compression therapy [21]. In spite of the paucity of scientific evidence, considering our clinical experience, we recommend local warm or cold applications at the level of the inflamed venous cord, based on the patient’s tolerability and perceived effectiveness, for their general physiological effects on inflammation and blood flow.

Compression stockings are frequently prescribed for patients with SVT, mainly in those with concomitant varicose veins. Only one randomized controlled trial has been performed investigating the use of compression stockings, published in 2014: 80 patients with SVT, treated with prophylactic doses of LMWH, were randomized to wear compression stockings or no compression. The group wearing compression stockings had no difference in pain intensity, need of analgesics, or intensity of local symptoms, but had better thrombus regression [45]. Therefore, in our opinion, compression stockings could be prescribed for patients with SVT, more so in those with varicose veins, if the patients are willing to wear them. 

Topical NSAIDs are an efficient way to provide symptomatic relief, both in low-risk SVT, only symptomatically managed, and in high-risk patients [46]. Heparin gel formulations, topically applied, seem to be also an effective treatment to alleviate local signs and symptoms, simultaneously improving local microcirculation [29,47]. 

All of these locally applied treatments are important, as SVT is frequently a very symptomatic disease and oral NSAIDs would amplify the hemorrhagic risk of a concomitant systemic anticoagulant therapy.

### 5.3. Role of Surgery in SVT

Invasive treatment of incompetent veins in the superficial venous system, either through surgical or endovascular methods, should be considered after confirming the presence of pathological reflux via duplex ultrasound. However, these procedures should be recommended only after at least three months from the acute thrombotic event. Although widely practiced, this approach lacks evidence, being more of a consensus based on experts’ opinions [4,22].

In general, based on the evidence available at this moment, surgery should be complementary to anticoagulation in the treatment of SVT, as it was proved to be effective in reducing SVT extension and recurrence but failed to lower the incidence of subsequent VTE [22,48]. Casian et al. also published a study that demonstrated that surgery fails to reduce the incidence of VTE compared to anticoagulation alone in the acute setting [49].

Nevertheless, the experts propose that phlebectomy alone, when available, could be indicated in patients with limited involvement of varicose tributaries or with very limited involvement of the saphenous trunk [22,49].

### 5.4. Suggestions Regarding Treatment Options in Low-Income Countries

We agree that low-income countries may face challenges in providing, on a large scale, the therapeutic options for SVT with more or less solid evidence, as outlined in different international guidelines, such as fondaparinux, rivaroxaban, or even LMWH. In such contexts, vitamin K antagonists (VKA) could serve as a viable alternative, although they are relatively under-researched. A 6-month follow-up study, which included 562 patients with SVT associated with varicose veins and excluded obese, aged over 70, and cancer-affected individuals, demonstrated that warfarin was superior to simple elastic compression or to saphenous ligature with regard to symptom relief and thrombotic extension [48]. Certainly, besides VKA, interventions for symptomatic relief such as extremity elevation, warm or cold applications, topical NSAIDs, heparin gel, or compression stockings may also provide valuable benefits.

## 6. Superficial Vein Thrombosis in Pregnancy

SVT is a relatively frequent problem affecting pregnant women (SVT has a prevalence of 0.1% in pregnancy) and it has an approximately two-fold higher risk of venous thromboembolic complications compared to non-pregnant women [50]. These statements, together with very limited evidence regarding the optimal therapeutic approach, make the management of these cases very challenging.

The Balkan Working Group for the Prevention and Treatment of Venous Thromboembolism published a position paper in 2022 that agreed on some suggestions regarding the therapy of SVT in pregnancy, recommending a more aggressive approach than usual:

- the first line of treatment is LMWH (fondaparinux 2.5 mg daily only in allergic or intolerant patients; however, the use of fondaparinux in pregnant women is off-label, as this drug can cross the placenta and the experience using it is limited, especially in the first trimester; certainly, there is need for more data about the safety of fondaparinux during pregnancy) [51,52]
- in SVT located below the knee: prophylactic doses of LMWH for 6 weeks (for example, enoxaparin 40 mg SC daily or 60 mg SC daily in obese patients); in patients with additional thromboembolic factors, continuation after 6 weeks should be considered (known significant thrombophilia, immobilization, infections, inflammatory or autoimmune conditions, cancer, or history of VTE)
- in SVT located above the knee, >10 cm from the sapheno–femoral junction or below the knee, >5 cm from the sapheno–popliteal junction: intermediate dose of LMWH for the entire pregnancy period and 6 weeks post-partum (for example, enoxaparin 40 mg SC b.i.d.)
- SVT located <10 cm from the sapheno–femoral junction or <5 cm from the sapheno–popliteal junction: therapeutic dose of LMWH for the entire pregnancy period and 6 weeks post-partum (for example, enoxaparin 1 mg/kgc SC b.i.d.)
- elastic compression stockings could be a viable option, but mainly in cases of chronic venous insufficiency [53].

## 7. Atypical Localization in SVT

In hospitalized patients, superficial veins of the upper limbs are commonly affected, particularly in connection with the use of short peripheral venous catheters, peripheral vein infusions, or venipuncture [54]. SVT, as a complication of peripheral vein infusion, arises in 25–35% of hospitalized patients with peripheral vein catheters, carrying significant implications for the development of severe complications such as sepsis. 

The risk factors for peripheral vein infusion thrombophlebitis comprise the material of the catheter, the duration of catheterization, and local catheter-related infections. The optimal treatment for peripheral infusion-associated SVT is uncertain. General recommendations consist of cessation of infusion, removal of the catheter, and topic anti-inflammatory drugs, which can alleviate both pain and inflammation [23,55]. No data are available on the use of anticoagulants in peripheral infusion-associated SVT. 

SVT can also, albeit rarely, occur in other superficial veins, such as those in the abdominal wall, chest wall, penis (Mondor’s phlebitis), or neck [56]. Regarding therapeutic approaches for SVT in these locations, data are limited, and treatment is primarily aimed at managing symptoms. Hematologic investigations are generally unnecessary, and anticoagulation is not typically required due to the low risk of embolic complications and spontaneous resolution usually occurs within 6-8 weeks. [23,57]. However, given that these conclusions are drawn from case reports in the literature, we advocate for individualized therapeutic strategies tailored to the specific risk profile of each patient.

## 8. Superficial Vein Thrombosis: A Distinct Entity or a Manifestation of Venous Thromboembolic Disease Spectrum

The scientific view of SVT has evolved over time, along with the technical improvements of duplex ultrasound. Initially viewed as a benign condition and without robust data about its epidemiology, diagnostic methods, prognosis, and evidence-based therapeutic approach, nowadays it has become increasingly clear that SVT may be a manifestation of a systemic propensity for thrombosis, with a significant risk of concurrent or recurrent thromboembolic events. The unfolding of this concept is also reflected by changes in terminology. Although originally mentioned as ‘thrombophlebitis’, reflecting a more pronounced inflammatory component in its pathophysiology, it is currently mentioned more as ‘superficial vein thrombosis’, recognizing the importance of a generalized predisposition to thrombosis [58,59].

Imaging screening of the entire lower limb venous system is crucial once SVT is diagnosed, as concomitant DVT is often identified and changes the therapeutic management; likewise, there should be a high index of suspicion for PE. Di Minno et al. realized a large meta-analysis on 22 studies, including 4300 patients with SVT, and found concomitant DVT in 18% and concomitant PE in 6.4% of the cases [2]. Other studies, with significant numbers of patients, found the prevalence of concomitant DVT/PE ranging from 18% to 24.6% and 3.9% to 6.8%, respectively [6].

Various epidemiological studies showed that the incidence of subsequent DVT or PE in the first three months after SVT is significant and comparable to the incidence of the same events after primary DVT, emphasizing the idea of a hypercoagulable state and the need for anticoagulants for a period of time [58,60,61]. In the POST study, 844 patients with SVT of at least 5 cm in length were followed for 3 months. Despite 90.5% of the included subjects receiving anticoagulant treatment, 2.8% developed subsequent DVT, 0.5% developed subsequent PE, 3.3% experienced extension of SVT, and 1.9% experienced symptomatic recurrence of SVT. This analysis also identified certain risk factors for subsequent appearance of VTE events: history of DVT/PE, malignancy, male sex, and non-varicose veins [61]. Likewise, a cross-sectional, retrospective study conducted by Bell et al. aimed to investigate SVT complications within the first year: the combined prevalence of subsequent DVT or PE was 6.6%. Despite the 1-year surveillance period, 92% of DVT/PE occurred in the first 3 months. A multivariate analysis identified three risk factors for PE/DVT: the presence of an indwelling venous catheter within 30 days prior to SVT, history of malignancy with specific treatment in the past year, and non-surgical trauma occurring within 7 days prior to SVT episode [62].

**Figure 1:**
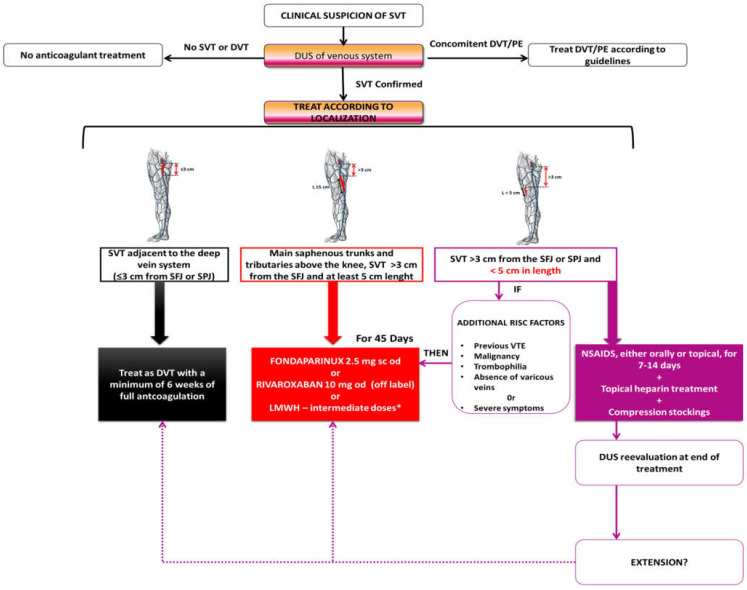
Proposed practical recommendations for clinical practice, adapted from [4,22,43,53]. * Intermediate doses of LMWH: Dalteparine 100 IU/Kg sc od or 5000 IU sc twice daily; Enoxaparine 40 mg sc twice daily; Nadroparine 2850–5700 IU sc od; Tinzaparine 4500 IU sc twice daily or 9000 IU sc od. DUS—duplex ultrasound, DVT—deep vein thrombosis, NSAID—non-steroidal anti-inflammatory drugs, od—once daily, PE—Pulmonary embolism, sc—subcutaneous, SVT—superficial vein thrombosis, SFJ—sapheno–femoral junction, SPJ—sapheno–popliteal junction.

Further studies are required, but as in the case of DVT or PE, hypercoagulability screening after a solitary episode of SVT is low yield; however, the experts recommend that screening for hypercoagulability and for malignancy is advised when SVT is recurrent or appears in non-varicose veins or in the absence of venous insufficiency [21] (Figure 1).

## 9. Conclusions

In conclusion, SVT is increasingly recognized as a marker of systemic tendency to thrombosis with significant implications regarding the recurrence and development of more serious thromboembolic events such as DVT or PE. The risk of these complications seems to be particularly elevated within the first three months following a SVT diagnosis, especially in patients with certain risk factors, such as male sex, history of DVT/PE, malignancy, non-varicose veins, a recent indwelling venous catheter, and non-surgical trauma within 7 days prior to the SVT episode.

Despite the limitations of studies on anticoagulation for SVT, evidence indicates that SVT is an independent contributor to VTE risk, requiring diligent management. The recognition that isolated SVT frequently coexists with DVT highlights the critical importance of systematic lower-limb DUS in its evaluation.

Anticoagulant therapy is currently considered a central pillar in SVT management, playing a crucial role in preventing early proximal extension and recurrent or subsequent VTE, although treatment strategies must be individualized based on the localization and thrombotic risk profile of the patient. While the existing guidelines offer valuable information, further consensus and research are needed to provide uniform, solid evidence-based recommendations. 

## Data Availability

The original contributions presented in the study are included in the article, further inquiries can be directed to the corresponding authors.
